# The effects of brodifacoum cereal bait pellets on early life stages of the rice coral *Montipora capitata*

**DOI:** 10.7717/peerj.13877

**Published:** 2022-08-16

**Authors:** Alexandria L. Barkman, Robert H. Richmond

**Affiliations:** Kewalo Marine Laboratory, University of Hawaii at Manoa, Honolulu, Hawaii, United States

**Keywords:** Coral reproduction, Pesticides, Brodifacoum, Rodent eradication

## Abstract

Midway Atoll in the Northwestern Hawaiian Islands is home to ground nesting birds that are threatened by invasive mice. Planned rodent eradication efforts for the island involve aerial application of cereal bait pellets containing the chemical rodenticide brodifacoum. Given the nature of the application method, drift of cereal bait pellets into the coastal waters surrounding Midway Atoll is unavoidable. To understand whether cereal bait pellets impact marine invertebrates, gametes and larvae of the reef-building coral *Montipora capitata* were exposed to brodifacoum, cereal bait pellets containing brodifacoum, and inert cereal bait pellets without the rodenticide. Fertilization success and larval survival were assessed at nominal brodifacoum concentrations of 1, 10, and 100 ppb. Fertilization success decreased by 15% after exposure to 100 ppb brodifacoum solutions. Larval survival was not reduced by exposure to brodifacoum solutions. Cereal bait pellets containing brodifacoum reduced fertilization success at 10 ppb brodifacoum in 0.4 g per L pellet solutions by 34.84%, and inhibited fertilization at 100 ppb brodifacoum in 4 g of pellet per L solution. Inert cereal bait pellets had similar effects, reducing fertilization success at 0.4 g of pellet per L by 40.50%, and inhibiting fertilization at 4 g per L pellet solutions. Larval survival was reduced by >43% after prolonged exposure to 4 g per L pellet solutions. The highest concentration used in this study was meant to represent an extreme and unlikely condition resulting from an accidental spill. Our findings indicate large amounts of cereal bait pellets entering the coastal environment of Midway Atoll, if occurring during a coral spawning event, would reduce coral reproduction by decreasing fertilization success. It is difficult to know the ecologically relevant concentrations of cereal bait pellets in coastal environments due to unavoidable bait drift after land applications, but results indicate small amounts of pellet drifting into coastal environments would not severely reduce coral reproductive capacity. Best management practices should consider known coral reproductive periods when scheduling applications of pellets on tropical islands to reduce the risk of negative impacts of large-scale accidents on corals.

## Introduction

Coral reefs are among the most biologically productive and diverse ecosystems in the world (Reaka-Kudla, Wilson & Wilson, 1997; Hoegh-Guldberg, 2014; Birkeland, 2015). They support the lives of millions of people through economic, recreational, ecological, and cultural services. In Hawai’i coral reefs are valued at '9.7 billion, including the services provided by protection of the shoreline, fisheries support, and '363.5 million in reef related tourism activities a year (Bishop et al., 2011). Corals are also culturally important in Hawai’i. According to the Hawaiian creation chant, the Kumulipo, the coral polyp is considered to be the basic unit of life (Liliuokalani, 1897).

Despite their importance, coral reefs are declining globally due to the compound effects of global stressors like ocean acidification, and local stressors including terrestrial runoff (Sweatman, Delean & Syms, 2011; Hughes et al., 2017; Greer et al., 2020). Terrestrial runoff can result in the introduction of toxicants such as pesticides to the marine environment, leading to decreased survival of early life stages of coral (Lewis et al., 2016; Flores et al., 2020). Coral reef persistence and recovery from stress depends on successful reproduction (Richmond, Tisthammer & Spies, 2018), however early life stages of many marine organisms are often the most sensitive to toxicants (Reichelt-Brushett & Harrison, 1999). Marine larvae, including coral, depend on chemical cues during substrate selection and the induction of metamorphosis (Freckelton, Nedved & Hadfield, 2017). Reef persistence and recovery is dependent in part upon dispersal and arrival of competent planula larvae from nearby reefs (Gouezo et al., 2019). Thus, pristine remote reefs, relatively free from high anthropogenic disturbance, are of particular importance since they can act as a larval repository providing seed for other areas of the ocean.

Many remote islands have suffered accidental introductions of invasive species that reduce island biodiversity, threaten persistence of critical species, and alter ecosystem function (Angel, Wanless & Cooper, 2009; Bolton et al., 2014; Shiels et al., 2014; Towns, Atkinson & Daugherty, 2006; Graham et al., 2018). Invasive rodents threaten bird populations around the world, including on Gough Island in the Southern Atlantic, multiple islands in the Southern Ocean, and Midway Atoll in the Northwestern Hawaiian Islands (Angel, Wanless & Cooper, 2009; Wanless et al., 2009; Jones et al., 2021; Work, Duhr & Flint, 2021). Introduced omnivorous rodents have wiped out native plant species and have been documented preying on bird eggs, chicks, and adult seabirds (Raine et al., 2020; Jones et al., 2021). To combat accidental introductions, rodent eradication efforts require swift removal of the invasive species, continued monitoring for remaining rodents, and prevention of reintroductions. Aerial applications of chemical rodenticides have been increasingly used since the 1960s to eradicate rodent populations on islands, most commonly in New Zealand (Towns & Broome, 2003). Rodents have been eradicated from at least 284 islands worldwide, but efforts have been less successful on tropical islands compared to temperate islands (Howald et al., 2007; Keitt et al., 2015).

Midway Atoll is located in the central North Pacific Ocean, and is part of the Papahānaumokuākea Marine National Monument. Midway Atoll is culturally important to Native Hawaiians, originally known to Hawaiians as Kuaihelani which means “backbone of the heavens”, referencing the reflection of the island in the sky (Kikiloi, 2010). Rats and mice were inadvertently brought to Midway Atoll around 1943 while it was an active military base. Rats were successfully eradicated in 1996; however, mice remain on the island. Midway Atoll is also home to the largest breeding colony of Laysan Albatross in the world (Harrison, 1990). Trail cameras have observed mice attacking adult albatross on Midway Atoll during egg incubations, which can lead to death or severe injury of the nesting birds (Work, Duhr & Flint, 2021). In light of this threat, mouse eradication efforts are proposed for Midway Atoll using three aerial applications with additional hand broadcasts of cereal bait pellets containing the rodenticide brodifacoum. Efforts are planned for the summer when fewer seabirds are present on the island, and the vegetation food source for the mice is the lowest due to the dry season (United States Fish & Wildlife Service, 2019).

Eradication efforts are expensive and require careful planning to minimize impacts on nontarget organisms. Cereal bait pellets containing chemical rodenticides are used to target specific organisms. Prior to 2007, aerial application of cereal bait pellets had been used in 76% of the areas targeted for rodent eradication, with brodifacoum being the most common active ingredient (Howald et al., 2007). Eradication efforts using aerial or hand broadcast application of chemical rodenticides over large areas have been restricted to isolated areas to minimize risk of human exposure. Brodifacoum is a second-generation anticoagulant rodenticide with highly lipophilic properties that is more potent and longer lasting than first-generation anticoagulants such as warfarin (Leck & Park, 1981; World Health Organization & International Programme on Chemical Safety, 1995; Howald et al., 2007).

While aerial application of cereal bait pellets occurs exclusively over land to target rodents, there are also unavoidable and small amounts of pellets that are blown or drift into the coastal waters. Effective operations will cover the entire island with pellets up to the water line to ensure coverage of the entire habitable area of the target species. Thorough coverage, however, also leads to inevitable contamination of pellets to the ocean through with tidal changes, as observed on Palmyra Atoll (Engeman et al., 2013). Additional runoff from land or accidental drops of pellets over water may introduce pellets to marine ecosystems. Surveys after use of pellets to eradicate rats found small amounts of brodifacoum in fish and shellfish sampled around the island briefly after application of pellets on land (Masuda, Fisher & Beaven, 2015). Brodifacoum was also detected at low levels (between 0.0034 and 0.0112 mg per kg) in fish collected in a lagoon on Wake Atoll 3 years after pellet applications (Siers et al., 2020).

Non-target mortality after applications have been noted, and often include birds and mammals (Pitt et al., 2015; Ogilvie et al., 1997). Birds may face secondary exposure threats from eating insects that consume bait pellets (Ogilvie et al., 1997; Howald et al., 2010). Insects may scavenge for bait pellets, and can contain 1 ppb brodifacoum for weeks after consumption ceases (Brooke et al., 2013). Ninety percent of slugs sampled after a brodifacoum application contained brodifacoum residue up to 0.77 mg per kg (770 ppb), highlighting a potential secondary exposure for other animals (Alomar et al., 2018). However, it is possible snails and insects will not eat the pellets, or may metabolize and excrete brodifacoum rapidly before birds can be exposed by eating them (Brooke et al., 2011). Rats were found to excrete 14–22% of the brodifacoum they ingested, highlighting another potential environmental pathway of brodifacoum to non-target organisms or the marine environment after a rainfall (Fisher, 2009). While excretion in mice has not been studied, similar life history and feeding strategies shared by rats and mice make excretion of brodifacoum by mice possible.

Extreme examples of pellets accidentally spilled into aquatic environments have been reported. A helicopter transporting pellets for an eradication effort accidentally dropped 700 kg of cereal bait pellets containing 20 parts per million (ppm) brodifacoum into a lake in New Zealand. There was no brodifacoum detected in water or soil samples after the incident (Fisher et al., 2012). The only documented case of direct introduction of cereal bait pellets containing brodifacoum to the marine environment occurred after a road transport accident resulted in twenty tons of bait pellets on the shore of a beach in southern New Zealand (Primus, Wright & Fisher, 2005). Visible residue of pellets persisted for 1 week, and it is believed that a significant portion of the brodifacoum remained adsorbed to bait particles that accumulated on the seabed. Although dead birds and seals were found in the area after the spill, no signs of brodifacoum toxicity were detected in these organisms. Mussels were found to contain 0.41 ppm brodifacoum the day after the spill, but concentrations decreased quickly in mussels after exposure. Limpets and abalone also had detectable concentrations of brodifacoum for 80 to 191 days after the spill, most likely due to continued exposure to brodifacoum through sediment consumption, and the long half-life of brodifacoum. It was determined that the spill effects were mostly contained to a 100-m^2^ area. Brodifacoum is nearly insoluble in water, so even large numbers of pellets in the water would not result in high concentrations of brodifacoum in the water. The concentrations of brodifacoum in seawater samples were below the minimum detectable limit of 0.02 ppb by 36 h after the accidental spill. However, residues in shellfish persisted for 31 months after the incident (Primus, Wright & Fisher, 2005).

Few laboratory studies have tested the effects of brodifacoum or cereal pellets containing brodifacoum on non-target organisms (Brooke et al., 2013; Brooke et al., 2011; Alomar et al., 2018). To date, no studies have been conducted on marine invertebrates such as corals. The back reef of Midway Atoll has high coral cover, and is dominated by *Montipora spp* (Kenyon, Wilkinson & Aeby, 2010). *Montipora capitata* is a broadcast spawning species that is widely distributed through the Main Hawaiian Islands, Northwestern Hawaiian Islands, and throughout the Pacific and Indian Oceans (Vroom & Braun, 2010; Veron et al., 2016), and whose reproductive peak coincides with the planned mouse eradication project on Midway. Therefore, the potential impact of rodenticide contamination on the reproductive success and early life stages of this dominant coral species are of serious concern. To test the potential effects of rodent eradication efforts on early life stages of corals, *M. capitata* gametes and larvae were exposed to brodifacoum, brodifacoum treated cereal bait pellet, and inert cereal bait pellet solutions in the laboratory. We hypothesized that exposure to brodifacoum, brodifacoum cereal bait pellets, and inert cereal bait pellets would decrease fertilization success and larval survival in *M. capitata*.

## Materials and Methods

### Solution preparation

Cereal bait pellets containing the active ingredient brodifacoum with the same formulation as those proposed for Midway Atoll mouse eradication efforts were made by Bell Laboratories and supplied by Island Conservation, the organization that has been contracted conduct mouse eradication efforts. These pellets are registered with the U.S. Environmental Protection Agency as Brodifacoum-25D Conservation, formulated for use in dry island climates for conservation purposes (US EPA registration number 56228-37). These brodifacoum pellets contained 0.0025% brodifacoum by weight along with nondisclosed, nontoxic components including oats and a green dye. Inert pellets were made with identical components without the addition of brodifacoum. Both pellet types were cylindrical with a diameter of 1.3 centimeters (cm), length of 0.72 cm, and weight of 1.76 grams (g).

Cereal bait pellets were crushed to a powder and homogenized using a mortar and pestle before being added to solutions. Small fractions of pellets powder were added to each jar or bowl as a powder. The amount of pellet powder used for target concentrations of brodifacoum was calculated based on stated brodifacoum content percent by mass of brodifacoum in pellets (0.0025%). The control treatment was 0.5 micron filtered seawater (FSW). The brodifacoum pellet treatments used were; 1 part per billion (ppb) nominal brodifacoum in 2 milligrams (mg) of crushed brodifacoum pellet in 50 mL of FSW (Low Brodifacoum Pellet), 10 ppb brodifacoum in 20 mg of crushed brodifacoum pellet in 50 mL of FSW (Medium Brodifacoum Pellet), and 100 ppb brodifacoum in 200 mg of crushed brodifacoum pellet in 50 mL of FSW (High Brodifacoum Pellet) (Table 1). Inert Pellet solutions used were 2 mg of inert pellet in 50 mL FSW (Low Inert Pellet), 20 mg of crushed inert pellet in 50 mL of FSW (Medium Inert Pellet), and 200 mg of pellet in 50 mL FSW (High Inert Pellet). Crushed pellet was added directly to glass jars containing 50 milliliters (mL) of 0.5 micron filtered seawater the day of each experiment to make brodifacoum treated pellet solutions and inert pellet solutions. Nominal concentrations of brodifacoum were not confirmed using analytical chemistry.

**Table 1:** Description of experimental treatments and replicates.

Treatment label	Treatment solution	Fertilization experiment replication	Larval survival experiment replication
DMSO control	0.00033% dimethyl sulfoxide (DMSO) in 0.5 micron filtered seawater (FSW)	*n* = 20 total over 4 nights 6 on June 2021 night 1, 6 on June 2021 night 2, 2 on July 2021 night 1, 6 on July 2021 night 2	*n* = 5 June 2021
1 ppb Brodifacoum	1 ppb brodifacoum and 0.0000033% DMSO in 0.5 micron FSW		
Medium Brodifacoum	10 ppb brodifacoum and 0.000033% DMSO in 0.5 micron FSW		
High Brodifacoum	100 ppb brodifacoum and 0.00033% DMSO in 0.5 micron FSW		
FSW control	0.5 micron filtered seawater		*n* = 10 5 reps in June 2021, 5 reps in July 2021
Low Inert Pellet	0.04 g per L of crushed inert pellet in 0.5 micron FSW		
Medium Inert Pellet	0.4 g per L of crushed inert pellet in 0.5 micron FSW		
High Inert Pellet	4 g per L of crushed inert pellet in 0.5 micron FSW		
Low Brodifacoum Pellet	1 ppb brodifacoum in 0.04 g per L of crushed pellet in 0.5 micron FSW		
Medium Brodifacoum Pellet	10 ppb brodifacoum in 0.4 g per L of crushed pellet in 0.5 micron FSW		
High Brodifacoum Pellet	100 ppb brodifacoum in 4 g per L of crushed pellet in 0.5 micron FSW		

For the brodifacoum only treatments, pure brodifacoum (CAS 56073-10-0; Sigma-Aldrich, St. Louis, MO, USA) was dissolved in dimethyl sulfoxide (DMSO), then used to create various concentrations of the active ingredient in FSW by adding 100 ppm brodifacoum stock solution directly to FSW in treatment containers. The use of DMSO followed the guidelines laid out by the Organization for Economic Cooperation and Development (OECD) (2019) for testing of chemicals using a solvent at a concentration lower than 0.1% v/v. DMSO was chosen as a solvent because it is naturally occurring in corals (Gardner et al., 2017), and has been found to be less toxic than other solvents for aquatic organisms (Hutchinson et al., 2006; Thong et al., 2013). The concentration of DMSO used in this study was less than the LOEC of 10 ppm DMSO in an anthozoan ecotoxicology experiment (Machado e Silva et al., 2021). The nominal concentrations tested were 1 ppb brodifacoum, 10 ppb brodifacoum, 100 ppb brodifacoum, and a 0.00033% DMSO control solutions (Table 1). All pure brodifacoum treatments contained 0.00033% DMSO or less.

### Fertilization assays

*M. capitata* corals release buoyant gamete bundles containing both eggs and sperm 0–3 days after the new moon May–August. Gamete bundles break apart minutes after release, with subsequent fertilization of eggs requiring outcrossing with sperm from other colonies (Kolinski & Cox, 2003). Gamete bundles were collected from *M. capitata* colonies located near Lilipuna Pier in Kānéohe Bay, O’ahu (21.42973 N, 157.79220 W). Field collection in this area has been conducted by the Richmond Lab from the Kewalo Marine Laboratory previously, and described in Damiani (2020). Mesh traps placed over full colonies concentrated all gametes released from a single colony into a floating container. Collected gametes were gently poured into separate bowls to keep bundles separated by source colony. Nine total gamete bundles from the same three source colonies were added to 50 mL solutions in 100 mL glass jars with Teflon lined caps. Experiments were conducted during four spawning nights: June 7th, June 8th, July 9th, and July 10th 2021 (Table 1). Six replicate assays per treatment were conducted each night on three of the four nights. Only two replicates were conducted on the night of July 9th due to fewer gamete bundles having been released (*n* = 6 per night on three nights, *n* = 2 on one night, 20 total replicates). Source colonies were not consistent across spawning nights. Bundles broke apart in solutions where fertilization occurred. Ten embryos per replicate (*n* = 20) were preserved in 10% formalin solution after 5 h to stop further development of embryos, then scored for fertilization success the next day. Fertilization assays using gamete bundles from more than one source colony are commonly conducted to evaluate the effects of putative toxicants on corals (Markey et al., 2007; Hédouin & Gates, 2013; Negri et al., 2005). Fertilization success was consistently high across treatment night in this experiment, but gamete quality and fertilization success is known to be variable (Hédouin & Gates, 2013; Miller et al., 2018).

## Larval development assays

Fifteen hours after fertilization assays were started, remaining intact larvae were transferred and rinsed gently in beakers containing FSW. Larvae from these beakers were transferred to 25 mL glass bowls containing ten larvae per replicate with 10 mL of FSW and monitored for 3 days.

## Larval survival assays

Extra gamete bundles collected from the same *M. capitata* colonies used for fertilization assays, additional gamete bundles collected from other traps, and gamete bundles collected from the ocean surface were added to a cooler containing two micron filtered seawater and allowed to fertilize. Seawater was changed three times with 0.5 micron filtered seawater 1 h after the addition of bundles to remove excess sperm. Seawater changes were completed once a day while fertilized gametes were undergoing embryogenesis and larval development.

Ten 1 week old larvae from the per replicate (*n* = 10 for pellet solutions) were added to 25 mL glass bowls containing 10 mL of the same treatment solutions used for the fertilization assays. Crushed pellet was added directly to the glass bowls prior to the addition of larvae with the same treatments of FSW Control, Low Brodifacoum Pellet, Medium Brodifacoum Pellet, High Brodifacoum Pellet, Low Inert Pellet, Medium Inert Pellet, and High Inert Pellet solutions. The pellet treatments were all compared to the FSW Control treatment. Larval behavior was monitored, and survival quantified daily for 3 days. Larvae were considered alive if they were swimming, had visible, moving cilia, or moved when lightly prodded with a pipette tip. Larval swimming behavior was assessed visually as either actively swimming (Yes), or not swimming (No).

Additionally, larval assays using 0.00033% DMSO Control, 1 ppb Brodifacoum, 10 ppb Brodifacoum, and 100 ppb Brodifacoum treatments were conducted (*n* = 5). Larval behavior was monitored, and survival quantified daily for 3 days and compared to the DMSO Control treatment. Larval survival assays are commonly conducted to evaluate the effects of putative toxicants on early life stages of corals because they are known to be more sensitive to stressors than adults (Reichelt-Brushett & Harrison, 1999; Markey et al., 2007; Flores et al., 2020).

## Statistical analyses

Fertilization experiments were conducted on four separate nights with six replicates per night during both June spawning events, six replicates during the second night of the July spawning, and two replicates on the first night of the July spawning due to low output. All results were combined for a total of 20 replicates. Standard least squares models were used to analyze the data in JMP Pro 16 with fertilization success as the role variable, treatment as a fixed effect, and date as a random effect. Dunnett’s multiple comparisons test was used to compare treatments to the control (FSW for pellet treatments, DMSO for brodifacoum treatments). Separate models were used to analyze the pellet treatments and the brodifacoum only treatments. Multiple pair-wise comparisons using Tukey’s HSD procedure was used to test for differences between pellet types (brodifacoum vs inert).

A single experiment with four replicates was run to monitor survival of larvae fertilized in treatment solutions. Data were not normally distributed, so the non-parametric Kruskal-Wallis and *post hoc* Steel test with control were conducted. The DMSO Control treatment was used as the control in the brodifacoum only experiments, and FSW Control treatment was used as the control for the pellet experiments for statistical analyses.

A larval survival experiment was conducted using brodifacoum only solutions (*n* = 5). Data were not normally distributed, so the non-parametric Kruskal-Wallis and *post hoc* Steel test with control were conducted. Behavior and survival were compared to the DMSO Control treatment in statistical analyses.

Pellet solution larval survival assays were run twice, with five replicates in June, and five replicates in July 2021. A standard least squares models was used with larval survival as the role variable, treatment as a fixed effect, and date as a random effect. Dunnett’s multiple comparisons test was used to compare treatments to the FSW Control treatment. Multiple pair-wise comparisons using Tukey’s HSD procedure was used to test for differences between pellet types. All data were analyzed using JMP Pro 16.

## Results

### Fertilization assays

#### Brodifacoum treatments

**Figure 1:**
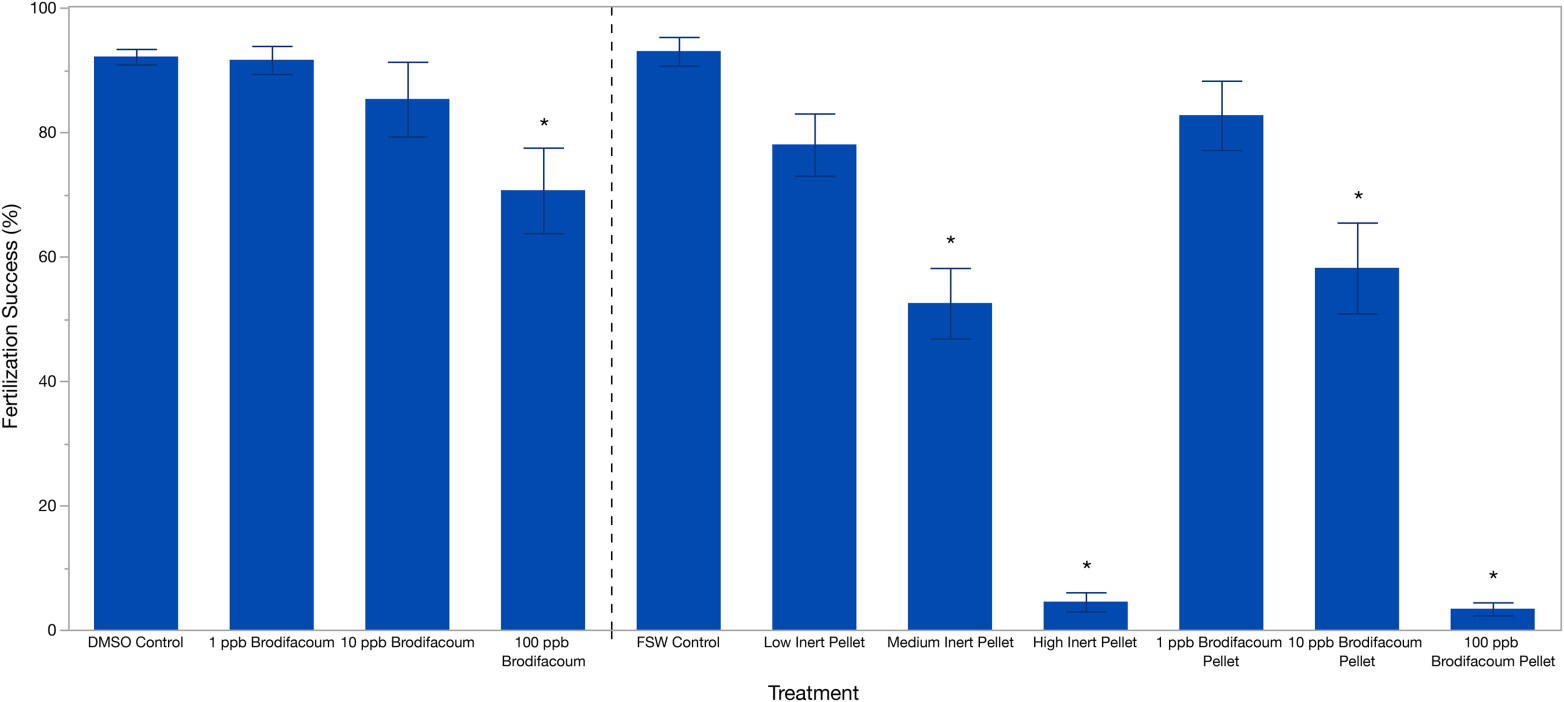
Fertilization success (%) of *M. capitata* gametes exposed to brodifacoum, cereal bait pellets containing brodifacoum, and inert cereal bait pellet solutions. The bars show mean +/− standard error (*n* = 20). Vertical dashed line used to separate experiments, consolidated into one graph. Significant differences (*p* < 0.05) compared to the relevant control (DMSO Control for brodifacoum treatments, FSW Control for all pellet treatments) indicated with an asterisk (*).

Brodifacoum only treatments were analyzed separately from the pellet treatments. Effect screening showed an effect of treatment (*p* = 0.0049), but no effect of date (*p* = 0.4912). Fertilization success was 92.13% ± 5.58 (mean ± S.D.) in the DMSO Control (Table 2). Fertilization decreased slightly with increased concentration of brodifacoum from 91.61% (±10.08) in 1 ppb Brodifacoum, 85.03% (±26.88) in 10 ppb Brodifacoum, and 70.64% (±30.75) in 100 ppb Brodifacoum treatments (Fig. 1). There was not a significant decrease in fertilization success in 1 ppb or 10 ppb Brodifacoum treatments compared to the DMSO Control. There was a significant decrease in fertilization success in the 100 ppb Brodifacoum treatment compared to the DMSO Control (*p* = 0.004). Fertilization success was high in the 100 ppb Brodifacoum treatment on June spawning night one (85%), June spawning night two (90%), and July spawning night one (85%), but low during the second July spawning night (32%).

**Table 2:** Average fertilization success (%) after 5 h. Average fertilization success and standard deviation of each treatment 5 h after gametes bundles were added to treatment solutions.

Label	Average fertilization success (%)	Standard deviation
DMSO control	92.13	5.58
1 ppb Brodifacoum	91.60	10.08
10 ppb Brodifacoum	85.30	26.88
100 ppb Brodifacoum	70.64	30.75
FSW control	93.00	10.31
Low Inert Pellet	78.00	22.38
Medium Inert Pellet	52.50	25.35
High Inert Pellet	4.50	6.86
Low Brodifacoum Pellet	82.70	24.9
Medium Brodifacoum Pellet	58.16	32.67
High Brodifacoum Pellet	3.37	4.71

#### Pellet fertilization assays

Effect screening showed an effect of treatment (*p* < 0.0001), but no effect of date (*p* = 0.3151) in the standard least squares model. Fertilization success was high in the FSW Control treatment at 93% ± 10.31 (mean ± S.D.) and decreased with increasing concentrations of both brodifacoum and inert pellets (Fig. 1, Table 2). Fertilization was inhibited in the high concentrations of both pellet types. Fertilization success was relatively high and not reduced in the Low Inert Pellet treatment at 78% ± 22.38, as well as the Low Brodifacoum Pellet treatment (82.70% ± 24.9). Fertilization was reduced in both the Medium Inert Pellet and Medium Brodifacoum Pellet treatments, as well as High Inert Pellet and High Brodifacoum Pellet treatments (*p* < 0.0001 for all). Fertilization success was 52.5% ± 25.35 in Medium Inert Pellet, and 4.5% ± 6.86 in the High Inert Pellet treatment. Fertilization success was 58.16% ± 32.67 in Medium Brodifacoum Pellet and 3.37% ± 4.71 in High Brodifacoum Pellet treatments. There were no significant differences between Low, Medium, or High treatments between pellet types (*p* > 0.97 for all comparisons).

### Larvae fertilized in treatments

Larvae fertilized in FSW Control and DMSO Control treatments appeared normal and swam normally. All treatments with pure brodifacoum in DMSO had high larval survival and normal swimming behavior compared to the controls. There were no larvae to observe in the High Inert Pellet or High Brodifacoum Pellet treatments due to the low fertilization success. Larvae fertilized in Low Inert Pellet, Medium Inert Pellet, Low Brodifacoum Pellet, and Medium Brodifacoum Pellet survived, appeared normal, and swam normally compared to larvae fertilized in FSW Control and DMSO Control treatments. There were no reductions in survival of larvae fertilized in treatments then moved to FSW compared to larvae fertilized in relevant controls (*p* > 0.2 for all comparison to controls). These results indicate that successfully fertilized embryos from treatments continued to develop normally in the absence of chemicals.

### Larval survival assays

#### Larvae fertilized in FSW, exposed to brodifacoum solutions

**Figure 2:**
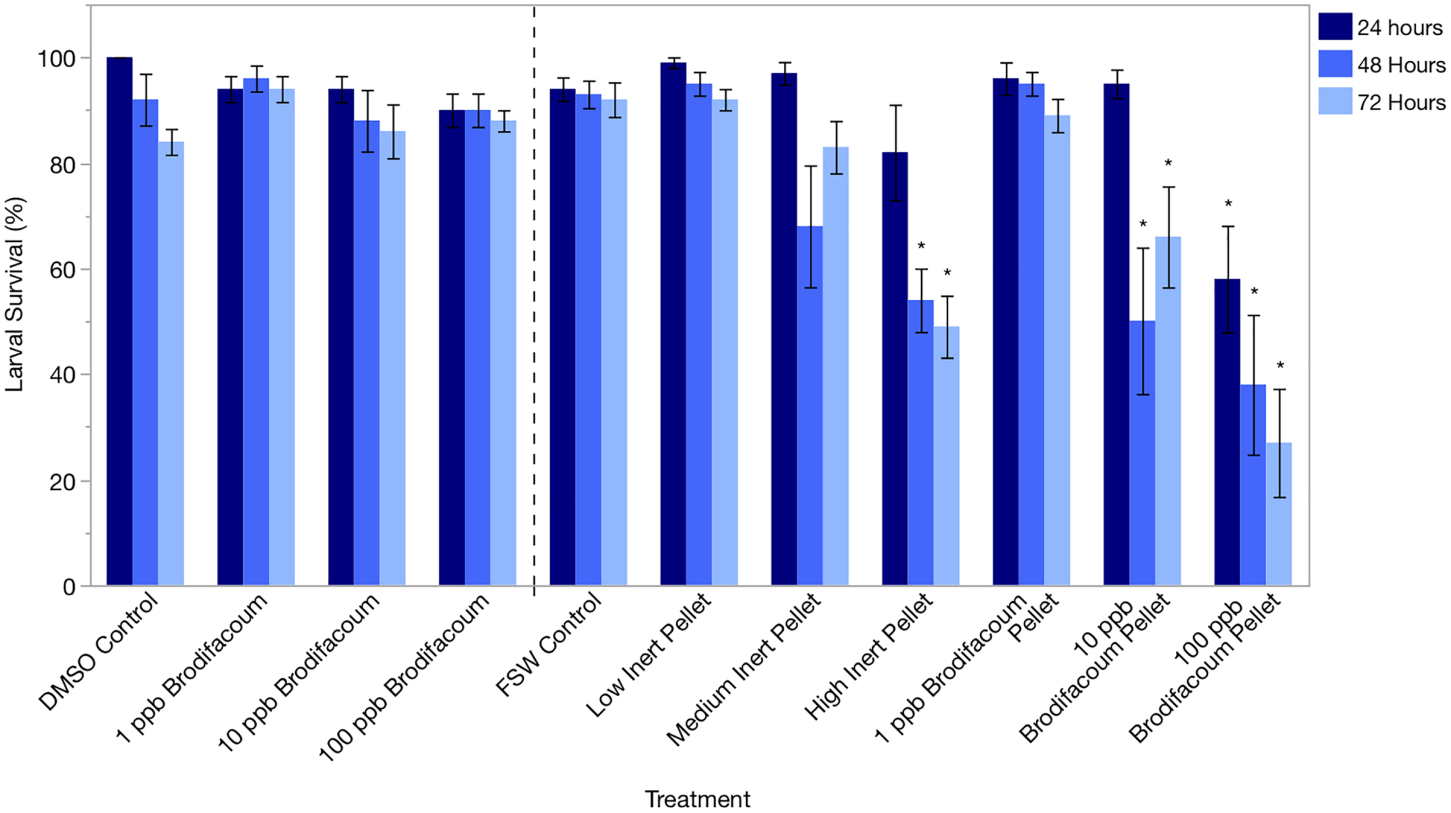
*Montipora capitata* larval survival (%) after 24 h, 48 h, and 72 h of exposure to brodifacoum, brodifacoum cereal bait pellet, and inert cereal bait pellet solutions. The bars show mean +/− standard error (*n* = 5 for brodifacoum treatments, *n* = 10 for pellet treatments). Vertical dashed line used to separate brodifacoum only experiments from pellets experiments, combined for of ease of viewing. Significant differences (*p* < 0.05) compared to the relevant control (DMSO Control for brodifacoum treatments, FSW Control for all pellet treatments) indicated with an asterisk (*).

Larval survival was high in the DMSO Control and 1, 10, and 100 ppb Brodifacoum treatments during the 3-day exposure. There was no decrease in larval survival in any treatment (Fig. 2, Table 3).

**Table 3:** Average larval survival (%) of larvae exposed to treatments for 1, 2, and 3 days.

Treatment	24 h		48 h		72 h	
	Average larval survival (%)	Std Dev	Average larval survival (%)	Std Dev	Average larval survival (%)	Std Dev
DMSO control	100	0	92.00	10.95	84.00	5.48
1 ppb Brodifacoum	94.00	5.48	96.00	5.48	94.00	5.48
10 ppb Brodifacoum	94.00	5.48	88.00	13.04	86.00	11.4
100 ppb Brodifacoum	90.00	7.07	90.00	7.07	88.00	4.47
FSW control	94.00	6.99	93.00	8.23	92.00	10.33
Low Inert Pellet	99.00	3.16	95.00	7.07	92.00	6.32
Medium Inert Pellet	97.00	6.75	68.00	36.45	83.00	15.67
High Inert Pellet	82.00	28.60	54.00	18.97	49.00	18.523
Low Brodifacoum Pellet	96.00	9.67	95.00	7.07	89.00	9.94
Medium Brodifacoum Pellet	95.00	8.50	50.10	43.84	66.00	30.26
High Brodifacoum Pellet	58.00	31.90	38.00	41.85	27.00	32.34

#### Larvae fertilized in FSW, exposed to pellet solutions for 3 days

**Figure 3:**
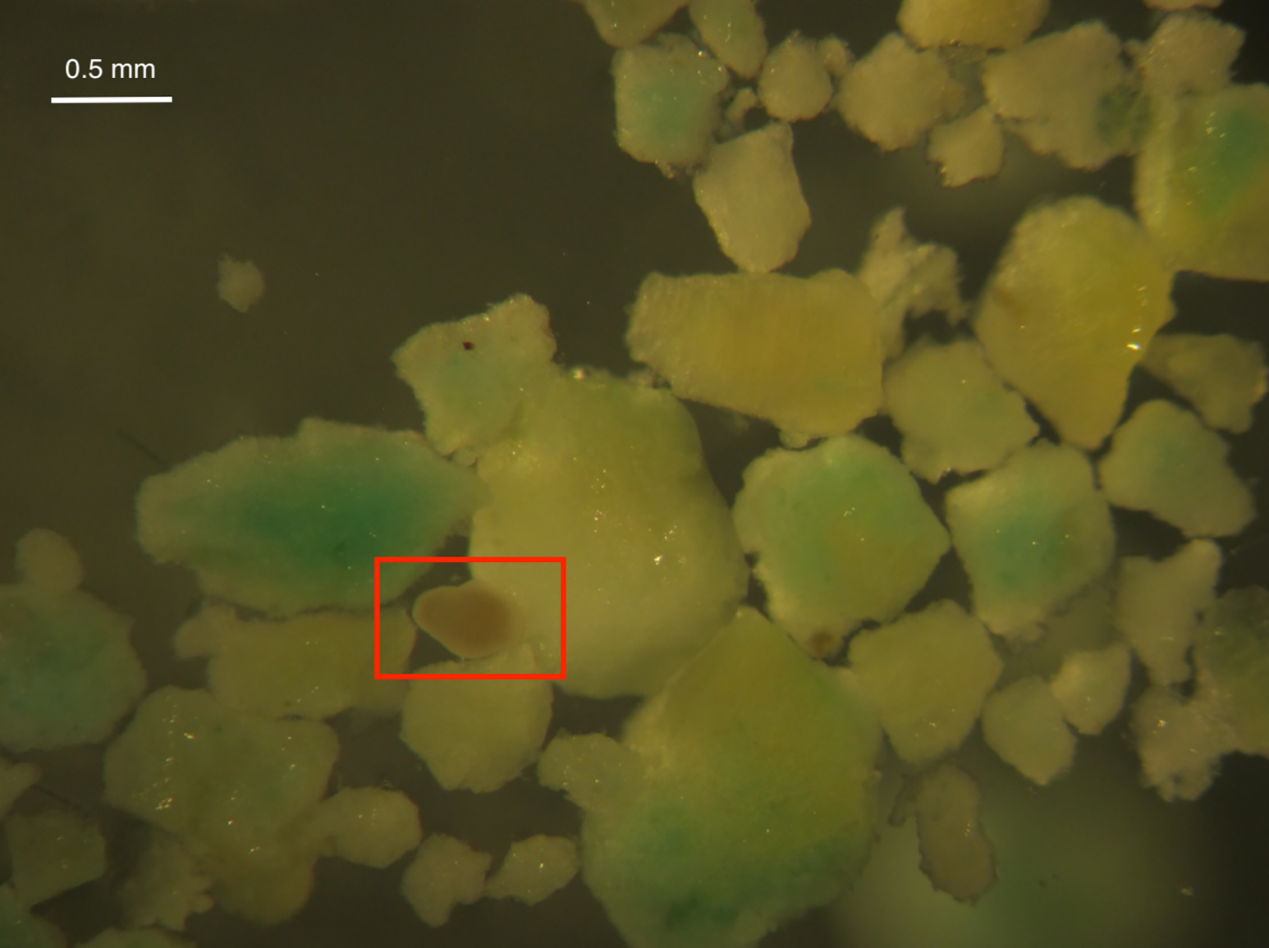
*M. capitata* larvae attached to pellet particles. An example of a *Montipora capitata* larva attached to a piece of crushed brodifacoum treated pellet. Larva is in the center of the rectangle.

Larval survival was high in all treatments after 24 and 48 h except for High Inert Pellet and High Brodifacoum Pellet treatments (Fig. 2). Larval activity was reduced in these treatments, and larvae appeared to be stuck to particles floating on the surface or at the bottom of the glass (Fig. 3). Larval survival was only significantly reduced in the High Brodifacoum Pellet treatment after 24 h (*p* = 0.0266). Survival was reduced to 82% ± 28.57 (mean ± S.D.) in High Inert Pellet, and 58% ± 31.9 in High Brodifacoum Pellet treatments after 24 h, then 54% ± 18.97 in High Inert Pellet and 38% ± 41.85 in High Brodifacoum Pellet treatments after 48 h. The reduction in larval survival was significant in High Inert Pellet (*p* = 0.0166) and High Brodifacoum Pellet (*p* = 0.0019) after 48 h. Larvae were counted as alive if they appeared intact and would swim when prodded lightly. Most larvae in both high pellet treatments were not moving on day 2. By day 3, most larvae in High Inert Pellet and High Brodifacoum Pellet treatments had degraded, and those remaining were not swimming. Larval survival was again only significantly reduced in the High Inert Pellet (49% ± 18.53) and High Brodifacoum Pellet (27% ± 32.34) treatments (*p* = 0.0013, *p* = 0.0024). The experiment ended after 3 days because most larvae were degraded in both the High Inert Pellet and High Brodifacoum Pellet treatments. Larvae in other treatments were not monitored daily after day 3. There were no significant differences between paired treatments of the same pellet amount between inert or brodifacoum pellet treatments.

## Discussion

Fertilization success was only reduced after exposure to pure brodifacoum solutions at a high concentration of 100 ppb brodifacoum. Brodifacoum has limited solubility in water and is unlikely to reach concentrations of 100 ppb in the field after dissolution of pellets or continued introduction of brodifacoum attached to soil through runoff. Pure brodifacoum solutions were used to increase understanding of potential impacts of the rodenticide on corals in a controlled environment. The use of DMSO to dissolve brodifacoum to allow for manipulations of brodifacoum concentrations in a controlled setting followed the guidelines laid out by the Organization for Economic Cooperation and Development (2019) for testing of chemicals. Operational use of brodifacoum would not include increasing solubility by dilution with DMSO. Brodifacoum is lipophilic and could bioaccumulate in marine organisms. Despite a lack of detection of brodifacoum in water samples, it does bioaccumulate in marine organisms after rodent eradication events at low levels of 0.273 ± 0.056 µg/g (mean ± SE) in hermit crabs and 0.143 ± 0.027 µg/g (mean ± SE) in black spot surgeon fish (Pitt et al., 2015). Corals have not been sampled after eradication events, so there is no documentation of bioaccumulation of brodifacoum in corals. Brodifacoum is not highly mobile in soil with a tendency to adsorb to soil and suspended particles in water (K_oc_ = 1.4 × 105). Brodifacoum has an estimated bioaccumulation factor of 570, indicating high potential for bioaccumulation in aquatic organisms. (National Center for Biotechnology Information, 2022). Bioaccumulation of brodifacoum in corals is possible because corals accumulate hormones and toxicants from the surrounding environment and through feeding (Armoza-Zvuloni et al., 2012; Yang et al., 2019). It is not known whether bioaccumulation of brodifacoum would result in health effects in adult corals. The concentrations used in this study were likely higher than those that would be expected to occur in nearshore environments, hence, it is believed that brodifacoum may not affect coral health at levels that nearshore colonies would encounter.

Fertilization success and larval survival were significantly reduced after exposure to 0.4 and 4 g per L solutions of both brodifacoum treated and inert cereal bait pellets. Larval survival was only significantly decreased after prolonged exposure to high concentrations of either inert or brodifacoum pellets. Larvae are made of mostly lipid, which may have contributed to the tendency of larvae to stick to pellet pieces floating on the surface or on the bottom of the glass bowl (Richmond, 1987). Brodifacoum is lipophilic, and may be easily absorbed by larvae upon contact with pellet particles. The pellet formulations were identical, other than the addition of brodifacoum to the brodifacoum pellets. In the absence of brodifacoum, we saw similar results among treatments, demonstrating that the reactivity is the result of the pellets themselves, and likely not due to brodifacoum exposure. Although fertilization was not significantly reduced in the low concentration of either pellet, fertilization success was decreased and more variable in the low treatments (S.D. > 22% compared to ~10% in controls). Variable fertilization success is regularly observed in broadcast spawning species between spawning events, and may be enhanced by pellet addition (Padilla-Gamiño & Gates, 2012; Hédouin & Gates, 2013).

The lowest pellet concentration used in this study was 40 mg of pellet per L, or approximately one pellet per 44 L. Pellet densities around islands after aerial application are variable (Engeman et al., 2013), but the concentration of pellets used in this study might still be higher than expected from accidental drift of pellets during aerial applications. The medium and high concentrations of pellet that led to reductions in fertilization are likely only representative of extreme accidents where large amounts of pellets enter an area with high residence time, or an accidental spill leads to pellets being dropped directly into the ocean.

Non-active ingredients of the pellets were not disclosed, but likely include oats and sugars based on available information on other pellet types for residential and commercial use. We hypothesize that the introduction of organic material from the pellets leads to bacteria proliferation that results in reduced dissolved oxygen. Hypoxia decreases fertilization and gamete formation in polychaetes (Shin et al., 2014), causes behavioral changes in coral larvae (Jorissen & Nugues, 2021), and decreases survival of adult corals in hypoxic zones (Altieri et al., 2017). The 2013 Honolulu Harbor Molasses Spill resulted in the deaths of thousands of fish and all corals within the Honolulu Harbor due to a steep drop in dissolved oxygen after 1,400 tons of molasses was spill in the harbor. Like most of the ingredients in the cereal bait pellets, molasses is nontoxic, yet still negatively impacted marine organisms at the high concentration that resulted from the spill (Hawai’i State Department of Health, 2013; Han & Singh, 2022).

Additionally, increased turbidity and decreased light availability due to suspended pellet particles could potentially impact coral survival. Cereal pellets did not dissolve fully in water, leading to cloudy appearance in high concentrations, and particles floating and sinking in test containers. Increased suspended and deposited sediments decrease health and survival of corals (Gilmour, 1999; Erftemeijer et al., 2012; Tuttle & Donahue, 2020). Large amounts of pellet entering the coastal waters may smother benthic organisms, physically sheer adult corals, or prevent larvae from detecting suitable settlement substrate.

## Management implications

Our results suggest that only large amounts of cereal bait pellets entering the nearshore waters after eradication efforts, if coinciding with coral spawning events, may affect the reproductive success of corals by reducing fertilization success. The chances of an extreme event taking place during the planned eradication efforts on Midway Atoll are low, but efforts to avoid peaks in coral reproductive periods will farther reduce potential risk to coral reproduction. In an abundance of caution, we recommend managers consider coral reproductive periods when planning the drops of pellets to allow time for dissipation of pellet material before a spawning event. Coral spawning events take place only a few times a year, so a missed spawning event could mean failed reproduction for a whole year. Broadcast spawning is the most frequent mode of reproduction in corals, and takes place predictably a couple times a year based on the season and phases of the moon (Richmond & Hunter, 1990). Risk of spills of large amounts of pellet to the marine environment could be reduced by using hand applications when possible. Aerial applications are necessary to cover all habitable space on most islands to ensure successful eradication.

Extreme events leading to accidental drops of large amounts of pellets into coastal zones with high residence time will likely inhibit survival of early life stages of corals, but these events are rare and unlikely. Rodent eradication efforts are carried about by trained professionals using best practices to avoid negative impacts on nontarget organisms. Successful eradication events have positive impacts on biodiversity and ecosystem health. Recovery of native species and bird populations have been observed after successful eradications (Jones et al., 2021). Additionally, recovery of terrestrial ecosystems leads to long term benefits to marine ecosystems (Kurle et al., 2021). Eradication of invasive species is a conservation priority to restore ecosystem functioning of both terrestrial and marine ecosystems (Graham et al., 2018).

### Future steps

Future studies should be carried out to determine if early life stages of other marine organisms and coral species are similarly impacted by brodifacoum and brodifacoum treated cereal bait pellets. *M. capitata* was selected due to its wide distribution, and results may be applicable to other broadcast spawning species. Pellets contain particles that both sink and float, making it unclear where the brodifacoum in the pellet may reside once it enters the ocean. Larval settlement may be impacted if larvae are unable to find accessible substrate. Swimming larvae may come in contact with pellet material that is floating in the water column. More studies should be done to assess whether treated or untreated cereal bait pellets impact coral larval settlement and metamorphosis. Adult corals should also be studied to determine the effects of cereal bait pellets containing brodifacoum on important habitat building species. Collection of marine invertebrates, including corals, after rodent eradication events using brodifacoum will help determine if brodifacoum is bioaccumulating in marine organisms after planned efforts on Midway Atoll as observed after efforts on Palmyra Atoll (Primus, Wright & Fisher, 2005).

## Conclusions

This was the first laboratory study testing the effects of brodifacoum on corals. Exposure to pure brodifacoum solutions did not decrease fertilization success or larval survival in *M. capitata* at ecologically relevant concentrations. Since brodifacoum does not readily dissolve in water, it is unlikely that the concentration of brodifacoum in the water column would reach or exceed the concentrations used in this study. Cereal bait pellets used in rodent eradications efforts did decrease fertilization success and larval survival at high concentrations representative of extreme and rare accidents. These results suggest that brodifacoum alone is not a threat to fertilization success or larval survival in corals at environmentally relevant concentrations.

Best management practices would consider well documented coral reproductive events when planning rodent eradication efforts to avoid negative impacts on fertilization success. More research should be done on the effects of the brodifacoum treated cereal bait pellets used in this study on adult corals. Additionally, more research should be done on the effects of brodifacoum treated cereal bait pellets used for rodent eradication efforts on other marine invertebrates, especially those that consume sediment and may ingest pellet particles that fall into the ocean.

## Supplemental Information

10.7717/peerj.13877/supp-1 Supplemental Information 1 All *M. capitata* fertilization success (%) by spawning day. Fertilization success (%) of *M. capitata* gametes in brodifacoum in DMSO, inert pellet solutions, and brodifacoum pellet solutions.

10.7717/peerj.13877/supp-2 Supplemental Information 2 All larval survival (%) data by treatment. Fertilization success (%) of *M. capitata* gametes in brodifacoum in DMSO, inert pellet solutions, and brodifacoum pellet solutions.

## Additional Information and Declarations

### Data Availability

The following information was supplied regarding data availability: Fertilization data by night and larval survival data are available in the Supplemental Files.
